# Importance of planetary rotation for ventilation processes in deep elongated lakes: Evidence from Lake Garda (Italy)

**DOI:** 10.1038/s41598-019-44730-1

**Published:** 2019-06-05

**Authors:** Sebastiano Piccolroaz, Marina Amadori, Marco Toffolon, Henk A. Dijkstra

**Affiliations:** 1 Institute for Marine and Atmospheric research Utrecht, Department of Physics, Utrecht University, Utrecht, 3584 CC The Netherlands; 2 Department of Civil, Environmental, and Mechanical Engineering, University of Trento, Trento, I-38123 Italy

**Keywords:** Limnology, Physical oceanography, Planetary science

## Abstract

Ventilation mechanisms in deep lakes are crucial for their ecosystem functioning. In this paper we show the relevance of planetary rotation in affecting ventilation processes in relatively narrow, elongated deep lakes. Through a recent field campaign in Lake Garda (Italy), we provide explicit observational evidence for the development of lake-wide wind-driven secondary flows influenced by the Coriolis force in a narrow lake. The interpretation of these observations is supported by results from numerical simulations with a three-dimensional model of the lake. The results add an additional element, often neglected in narrow lakes, to be carefully considered when assessing the response of lakes to external forcing and climate change.

## Introduction

Deep lakes in the temperate region undergo long periods of thermal stratification during which a well-mixed layer, exchanging heat and gases with the atmosphere, forms above poorly mixed and nutrient rich deep waters. If thermal stratification weakens enough, surface and deep water mix promoting the redistribution of oxygen, nutrients, and energy along the water column, until possible homogenization (e.g.^1,2^). In this way, surface properties such as temperature and dissolved gas concentrations are imprinted on deep layers, in a process commonly referred to as deep ventilation (e.g.^3^).

Vertical transport and mixing are therefore key factors affecting the water quality and trophic status of lakes (e.g.^4–6^), including several chemical and biological processes (e.g.^7–9^). The occurrence of deep mixing events (DMEs), in particular, is highly relevant in those lakes that undergo irregular ventilation and less frequently than once a year (oligomixis), hence where deep layers remain isolated from the surface waters for a relatively long time. DMEs are primarily controlled by the thermal stratification of the water column and by the fluxes of heat and momentum at the lake-atmosphere interface (e.g.^1,10–12^). Consequently, any ongoing and future change in climate forcing able to alter the stratification and mixing regimes of a lake^13–15^ may seriously threaten the occurrence of DMEs and its ecological status.

Recent studies suggest that thermal stratification is expected to increase in response to a warming climate, with consequent inhibition of extent and frequency of convective DMEs in the future (e.g.^16–19^). The effect of an enduring decrease of DMEs is the progressive warming, depletion of oxygen, and increase of nutrient concentrations in deep layers, counterbalanced by a reduced fertilization of surface water. This scenario has already been observed in many temperate lakes (e.g.^20–22^), including several deep Alpine lakes. Examples are Lake Zurich^23^, Lake Constance^24,25^, Lake Lugano^26^, Lake Geneva and Lake Maggiore for which some future climate scenarios are also available^27,28^, and Lake Iseo, where the lack of DMEs over the last decades led to significant deoxygenation of deep layers^29–31^.

In deep lakes where water temperature is warmer than the temperature of maximum density, DMEs are typically associated with buoyancy-driven convective processes caused by the increase of surface water density (e.g., due to cooling of surface water during cold winters or to inflows of denser waters), determining the typical sawtooth structure of deep water temperature^32^, whereby gradual warming is punctuated by abrupt cooling events. However, convective mixing is not the only mechanism of ventilation in long, narrow, and deep lakes (such as alpine, glacial, and rift valley lakes, fjords, and river valley reservoirs^33,34^). Strong and sustained wind events can generate along-lake up-/downwelling episodes due to tilting of the thermocline and internal waves (e.g.^35–37^), or large scale overturning circulation reaching down to the lake bottom^38^.

Theoretical studies for constant density flows in rotating narrow, elongated, and deep basins have also shown that intense closed circulations in cross-sections orthogonal to the wind direction may develop due to the effect of the Coriolis acceleration^39–41^ and to the presence of lateral boundaries, with substantial up- and downwelling at the shore. Such flows were previously observed in numerical experiments in enclosed^42,43^ and semi-enclosed basins^44–46^, in the latter case particularly in the context of Regions of Freshwater Influence (ROFIs) where density-driven exchanges between estuary and ocean are relevant. However, they have never been explicitly measured in narrow deep lakes, where, in addition, their possible contribution to deep ventilation remains unexplored. In fact, after Ekman’s pioneering work^47^, this flow regime was traditionally observed and analyzed in large domains such as the ocean or large lakes^48^, while it was largely overlooked in relatively small enclosed basins^39,49^.

Using the deep and elongated peri-alpine Lake Garda (Italy) as case study, here we combine observations and numerical simulations to demonstrate that the combined effect of wind-induced circulation and planetary rotation is relevant for the ventilation of deep layers also in elongated lakes having a width on the order of a few km. These results add a new aspect of complexity when assessing the response of deep and narrow lakes to external forcing and climate change.

## Material and Methods

### Study area

**Figure 1:**
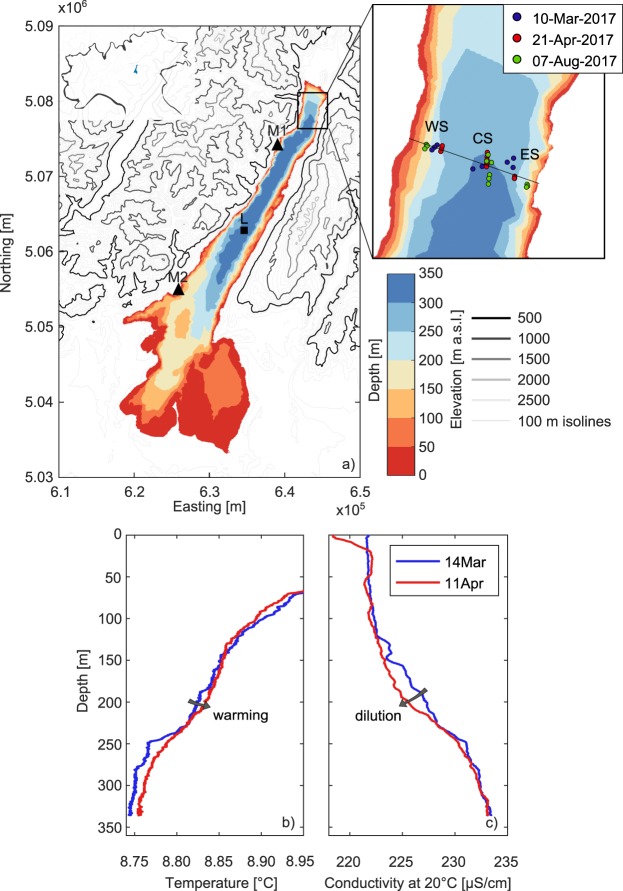
(**a**) Bathymetry of Lake Garda and topography of the surrounding region, with the location of Lake Garda in Northern Italy (inset), the reference transect with the monitoring stations (zoom-in view), and the meteorological (M1 and M2) and ARPAV monitoring (L) stations used in the analysis (black triangles and square, respectively). Vertical profiles of temperature (**b**) and conductivity at 20 °C (**c**) measured at the L station by ARPAV between March and April 2017 using a SBE-19plus SEACAT profiler.

With a surface area of 368 km^2^ and a water volume of 49 km^3^, Lake Garda is one of the largest lakes of the Alpine region and the largest in Italy. It is located in the northern part of the country (center at 45° 40′N and 10° 40′E), at the foot of the Alps (Fig. 1a). Its bathymetry is heterogeneous, being characterized by a narrow (average width 4 km) and deep (maximum depth 350 m) northern trunk enclosed between steep mountains, connected to a southern larger (maximum width 18 km) and shallower (average depth 65 m) basin lying in a flat plain. The lake has one main tributary, the Sarca River at the northernmost edge, and one emissary, the Mincio River at the southern shore. Owing to its large volume and relatively small inflow and outflow rates, the residence time is about 27 years, which is long compared to the other large sub-alpine lakes^50^.

The typical winds are characterized by coupled lake-valley breezes especially during warm-season clear-sky days^51^, while one-directional synoptic flows are frequent in wintertime often generating intense and long-lasting north-Föhn events^52^. In both seasons, winds in the northern part are predominantly directed along the major axis of the lake, due to the steep surrounding topography^53^.

The knowledge of the circulation in the lake is still at a first stage and current measurements are not available, but a preliminary modeling exercise^54^ provided a first description of the lake’s seasonal response to typical winds and suggested that Earth’s rotation could possibly affect transport processes in the lake.

The mixing regime of Lake Garda is classified as oligomictic with prolonged periods of incomplete mixing interspersed with occasional complete DMEs that involve the entire water column^20,55^. The temperature of the water is always above the temperature of maximum density, so that thermobaric effects^56^ do not affect the mixing regime of the lake. Salinity is low and its vertical gradients are small, thus playing secondary effects on the stability of the water column. A climate-induced change on DMEs has already been detected in the lake^57^. Complete (i.e., down to the bottom) buoyancy-driven convective DMEs were typically observed following particularly harsh winters causing surface cooling, the last event dating back to 2006. Since then, water temperature underwent a progressive increase along the whole water column, preventing the occurrence of significant convective DMEs.

However, the full (i.e., down to the bottom) vertical profiles measured by the Environmental Protection Agency of the Veneto Region (ARPAV) between March and April 2017 (Fig. 1b,c) suggest the existence of wind-driven flows as an additional, previously not recognized, ventilation mechanism. Visible warming of deep water down to 250 m depth accompanied by decrease in conductivity indicates that DMEs occurred after March 2017 and were most likely driven by the wind. In fact, the formation of progressively steeper profiles is not compatible with diffusive processes alone, while positive net heat flux to the lake after March (see Fig. S1 in the Supplementary Information) excludes the occurrence of buoyancy-driven DMEs. Building on this premise and expanding on the mentioned preliminary modeling analysis^54^, in the ensuing sections we provide evidence that wind-driven DMEs occur in Lake Garda and that these flows are modified by Earth’s rotation.

### Field campaign

Beginning in March 2017, a two-year monitoring program was established in Lake Garda^58^. As part of the monitoring activity, monthly high-resolution profiles of temperature and chlorophyll-a (besides other physical and turbulence related quantities) were measured with a loosely tethered, free-falling turbulence and CTD microprofiler (MicroCTD, Rockland Scientific International, RSI, Canada). The instrument is configured with a downward profiling speed of 0.7 ms^−1^ and has a maximum operational depth of 100 m. The thermistor and chlorophyll-a fluorometer (JFE-Advantech Sensors) have an accuracy of 0.01 °C and <1 ppb, a resolution of 0.001 °C and 0.01 ppb, and a sampling rate of 64 Hz and 512 Hz, respectively.

Three reference stations were selected during the monitoring campaign: Central Station (CS), located in the center of the narrow trunk at about 4 km from the northern edge of the lake, and two additional reference stations on the east (East Station, ES) and west (West Station, WS) sides of the CS. The three stations are aligned along a transect where the lake is ~2.5 km wide (Fig. 1a). Here we analyze the vertical profiles measured at the three monitoring sites after three significant synoptic northerly wind events on 10 March, 21 April, and 7 August 2017. All downcast profiles were taken at the same time of day, between 10:00 and 13:00 CET.

**Figure 2:**
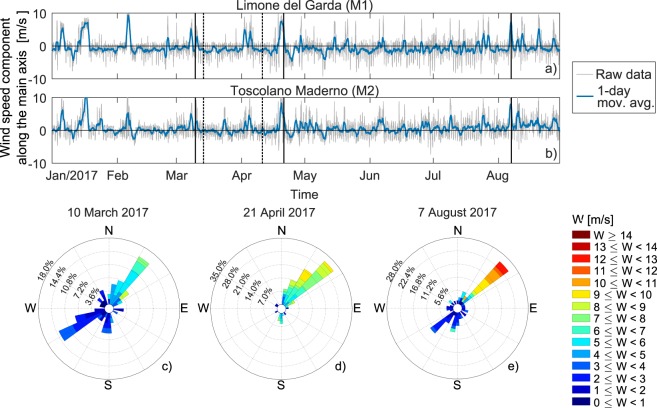
(**a**,**b**) Time series of wind speed component along the major axis of the lake (oriented about 30°E) measured at the meteorological stations in Limone del Garda-M1 (**a**) and Toscolano-Maderno-M2 (**b**, see Fig. 1a for M1 ans M2 stations location). Vertical continuous lines indicate fieldwork days, while vertical dashed lines indicate ARPAV monitoring days. (**c**–**e**) Wind roses for the three analyzed periods based on measurements collected at station M1 relative to a three-day period, including the fieldwork day and the two preceding days.

Wind speed and direction data were obtained from the meteorological stations operated by the Regional Meteorological Service of the Environmental Protection Agency of the Lombardia Region (ARPA Lombardia, Fig. 2). We analyzed the time series of wind speed measured at Limone del Garda and Toscolano-Maderno, respectively site M1 and M2 in Fig. 1a. Specifically, Limone del Garda (M1) was chosen as reference station, as it is located ~6 km south of the reference transect and 200 m from the shore.

### Numerical modeling

As a complement to *in-situ* measurements, numerical simulations of atmospheric and lake conditions were performed for the periods 3–11 March, 18–21 April, 1–15 August 2017, according to the field campaign dates (10 March, 21 April, and 7 August 2017).

#### Meteorological model

High-resolution fields of meteorological data were provided by the Weather Research and Forecasting (WRF) Model^59^, which was used to prescribe the surface forcing. The event-based simulations were performed according to the meteorological model setup used by^60,61^ to reproduce thermally-driven circulations in the lake area. The spatial domain covered the whole Lake Garda region and was composed of three two-way nested domains with 94 × 90, 112 × 97, and 73 × 106 cells, and grid spacing of 9, 3, and 1 km, respectively; 30 levels were used along the vertical. Initial and boundary conditions were provided by the 6-hourly National Centers for Environmental Prediction (NCEP) Final Operational Global Analysis data on 1-degree grids. The land use was defined from the Corine Land Cover dataset, having a spatial resolution of 100 m (provided by the European Environment Agency, http://www.eea.europa.eu). Spatial and temporal fields of the meteorological of the main variables simulated for the last domain (1 km spatial resolution) were saved at a temporal resolution of 15 minutes.

#### Hydrodynamic model

Lake thermo-hydrodynamics were simulated using the open-source modeling suite Delft3D^62^. The domain was discretized by a non-uniform, locally-orthogonal curvilinear grid staggered in space with 126 × 446 cells with average resolution of ~10 m, and 100 vertical layers with increasing thickness from 1 m at the surface to 25 m at the bottom. Initial conditions for DELFT3D were set as water at rest, horizontal water level, and spatially uniform vertical temperature profile according to the temporally closest profile measured at CS by the Environmental Protection Agency of the Province of Trento (APPA) (21 March and 5 July 2017, for April and August simulations respectively) or reconstructed from field campaign data (for March simulation). A 2-day spin-up period was shown to be long enough to reasonably remove the influence of initial conditions from hydrodynamic simulations of Lake Garda^54^, supporting the representativeness of the numerical results. Vertical eddy diffusivity and viscosity of the model were calculated with the *k* − *ε* turbulence model. The values of the horizontal viscosities and diffusivities where both set as 0.2 m^2^ s^−1^ according to the grid size^63^ and a preliminary sensitivity analysis. A computational time step of 60 s was used for March and April simulations, and 30 s for August simulation. The value of the main parameters of the model are summarized in Table 1.

**Table 1:** Hydrodynamic model set-up.

**Calibration parameters**				
Wall b.cs	free slip			
Bottom roughness (Chézy)	65 m^1/2^ s^−1^	(default)		
Wind drag coefficient *C*_*d*_ (for wind velocities *U*^a^)	0.0044 (1 m s^−1^)	0.0010 (5 m s^−1^)	0.0020 (20 m s^−1^)	
Turbulence closure	*k* − *ε*	Heat fluxes	*Ocean model*	
Horizontal eddy diffusivity	0.2 m^2^ s^−1^	Stanton number	1.3 × 10^−3^	(default)
Horizontal eddy viscosity	0.2 m^2^ s^−1^	Dalton number	1.3 × 10^−3^	(default)
**Event-based simulations**				
Event	Start time		End time	time step
March 2017	3 March 00:00		12 March 00:00	60 s
April 2017	18 April 00:00		22 April 00:00	60 s
August 2017	1 August 00:00		16 August, 00:00	30 s
**Numerical tracer simulations**				
Event	Instantaneous release time *t*_1_		End time *t*_2_	time step
March 2017	7 March 12:00		10 March 12:00	60 s
April 2017	19 April 00:00		22 April 00:00	60 s

^a^At 10 m above ground level.

#### Tracer experiment

**Figure 3:**
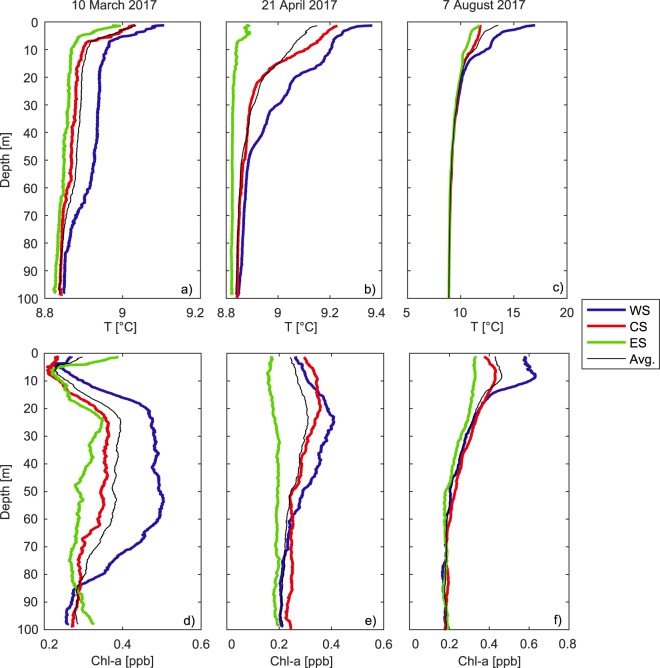
(**a**–**c**) Vertical profiles of temperature measured at the three sampling stations (ES, CS, and WS) in the three analyzed periods. (**d**–**f**) As in panels (a–c) but for chlorophyll-a. We notice that the depth of vertical profiles here and in Fig. 1 are different.

The hydrodynamic simulations described above included numerical tracer experiments. We quantified wind-induced ventilation by analyzing the evolution of a passive tracer initially distributed with constant concentration *C*_*surf*_ in the upper 50 m (based on the profiles in Fig. 3), while it was set to 0 mg/l along the remaining of the water column. We instantaneously released the tracer at the beginning of the wind event (*t*_1_) and let it be transported by the flow up to time *t*_2_ (see Table 1).

The volume *V*_*vent*_(*i*, *t*) of surface water ventilated in the deep lake from the beginning of the tracer experiment (time *t*_1_) until time *t* was estimated based on the mass balance of the tracer at each transverse model cross-section *i* (similar to^37^)

1 $${V}_{vent}(i,t)=\frac{{V}_{isopy}(i,t){C}_{isopy}(i,t)}{{C}_{surf}},$$

where *V*_*isopy*_(*i*, *t*) is the volume of water below a reference isopycnal surface at model cross-section *i* at time *t*, and *C*_*isopy*_(*i*, *t*) is its average tracer concentration. The above mass balance assumes that the tracer concentration of downwelled water is time-invariant and equal to *C*_*surf*_. The isopycnal surface was chosen such that the overall *V*_*isopy*_ was relatively small compared to the lake volume in order to be representative of the deep layer when the lake is calm and the isopycnals are horizontal. We chose the isopycnal such that *V*_*isopy*_ was about 18% of the lake volume (corresponding to the lake volume below nearly 200 m depth). *V*_*isopy*_ showed limited variability during the tracer experiment (±1% of lake volume), thus making it well delimited throughout the simulation.

In order to quantify the wind-induced ventilation in the lake, we calculated the ratio *η* between the ventilation volume reaching depths below the reference isopycnal surface and *V*_*isopy*_:

2 $$\eta (i,t)=\frac{{V}_{vent}(i,t)}{{V}_{isopy}(t)},$$

the sum of which over index *i* at a given time *t* can be interpreted as the efficiency of wind-driven ventilation of *V*_*isopy*_ at that time.

The tracer experiment was not performed for the wind event on August 2017 since it was not significant for the analysis of deep ventilation due to the strong thermal stratification of the lake in that period.

## Results

### Field campaign

The time series of the wind speed measured at M1 and M2 stations are plotted in Fig. 2a,b. The figure shows the wind speed component along the major axis of the lake, with positive values indicating wind from north-east and negative from south-west. Raw data are available at hourly interval, but for clearer reading the 1-day moving average is also shown. Strong and persistent wind events occurred during the fieldwork periods (vertical continuous lines), with significant events also in January and February, when the lake is typically weakly stratified^64^, and in early April between the two ARPAV monitoring days (vertical dashed lines), when the profiles in Fig. 1b,c have been measured.

For all analyzed periods the wind roses from M1 station in Fig. 2c–e), relative to the monitoring day and the preceding two-day time window, confirm that a synoptic south-easterly wind blew with wind speeds (mean ± standard deviation, computed from the raw hourly data) for the northeast quadrant of 5.3 ± 1.3 m s^−1^, 6.7 ± 1.6 m s^−1^ and 6.9 ± 3.8 m s^−1^ for the three events, respectively.

The lateral variation of the vertical profiles of temperature and chlorophyll-a measured at the three monitoring sites along the reference transect down to 100 m depth is shown in Fig. 3a–f. A significant lateral gradient is clearly visible, with water temperature and chlorophyll-a concentrations increasing from ES to WS along the upper 100 m of the water column. In March and April, the thermocline depth was deeper moving from east to west and the thermal stratification was stronger, while still being characterized by small vertical temperature gradients (i.e., on the order of 0.1 °C) according to the typical seasonal conditions. The temperature profiles at ES were nearly homogeneous (particularly in April), except for the upper ~1 m. The peak of chlorophyll-a concentration was deeper and more intense at WS, with high chlorophyll-a concentrations being well distributed over a wider range of depths. This was particularly visible in March, the profile at WS being characterized by a ~40 m-thick layer of nearly homogeneous chlorophyll-a concentrations, accordingly to the thick well-mixed layer (Fig. 3d). Similar considerations apply to the profiles measured in August, although the largest lateral differences were bounded within the upper ~3 m due to much stronger thermal stratification (Fig. 3c).

For all monitoring periods, the observations are consistent in showing a significant surface westward transport accumulating chlorophyll-a and warm water at station WS, which was not observed in the profiles measured in absence of persistent wind events in other periods of the year (see Fig. S2 in the Supplementary Information). This causes a closed circulation in the cross-section with up- and downwelling at the east and west shore of the lake, respectively. This pattern closely resembles the typical structure of wind-driven secondary flow in a rotating narrow and deep enclosed basin^39–41^, whereby the water circulation induced by a uniform and persistent wind (in this case blowing from the north) is deviated to the right (i.e., to the west) by the Coriolis force, generating a secondary closed circulation bounded by lateral shores.

### Numerical modeling

To validate the interpretation of the processes causing the lateral gradients described above, we performed numerical simulations of the lake thermo-hydrodynamics during the analyzed wind events. Here we show the simulations for the April event, which is chosen as a typical example of cold weather conditions and weakly stratified lake. Results for the March and August events are provided in the Supplementary Information.

**Figure 4:**
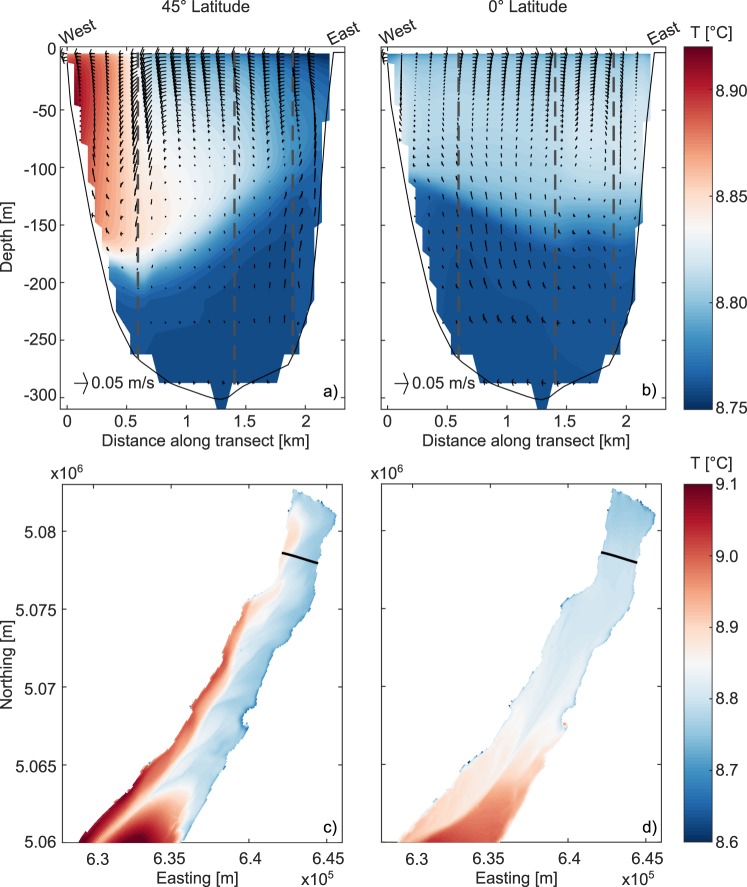
Results of thermo-hydrodynamic numerical simulations for the wind event on April 2017. Upper panels: water temperature and flow field (arrows) at the reference transect accounting for (**a**) and excluding (**b**) the Coriolis force. Vertical dashed lines indicate the approximate position of the monitoring stations. Lower panels: map of surface water temperature accounting for (**c**) and excluding (**d**) the Coriolis force. Black lines indicate the position of the transects shown in subplots (**a**,**b**).

Figure 4a shows the simulated water temperature along the model cross-section closest to the reference transect of the monitoring campaign. In addition to temperature, the simulated flow field is also shown. In order to improve representativeness and filter out possible short-time oscillatory effects, numerical results were averaged over a 6-hour period between 00:00 and 06:00 of 21 April, corresponding to the final part of the persistent wind event. Lateral temperature variability is fully consistent with observations in terms of both intensity and vertical extent. Detailed model validation is provided in the Supplementary Information, for both the hydrodynamic and the meteorological models (see Text S1). The simulated flow field confirms the existence of a significant westward transport within the upper 75 m associated with a secondary circulation involving almost the whole cross-section. The general pattern clearly matches that of wind-driven circulation influenced by Earth’s rotation, as hypothesized from the analysis of measured profiles (Fig. 3). In order to dispel any doubts in this regard, we performed a twin simulation keeping the same meteorological forcing and model setup, but neglecting the Coriolis acceleration. The numerical results are shown in Fig. 4b. The difference is glaring: in the non-rotating case the secondary circulation and hence the lateral temperature gradient are absent.

The same behavior is found in the maps of simulated surface water temperature shown in Fig. 4c,d. When the Coriolis effect is included, the entire narrow trunk of the lake is affected by a clear transverse flow that moves warmer surface water westwards while at the east shore it is replaced by upwelled colder deep water (Fig. 4c). In the non-rotating case this effect is absent, the water circulation being exclusively influenced by spatial heterogeneities of wind field, water temperature, and bathymetry (Fig. 4d).

**Figure 5:**
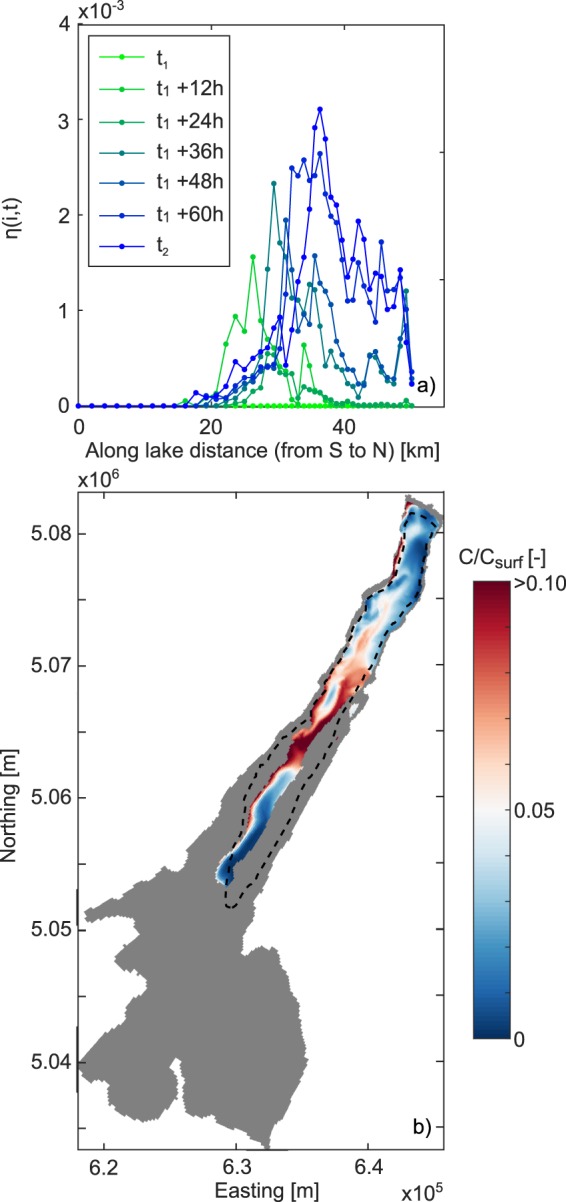
Results of the numerical tracer experiment for the wind event on April 2017. (**a**) Along-lake distribution of the normalized ventilation volume *η* from the release of the tracer (*t*_1_) to the end of the experiment (*t*_1_). (**b**) Map of tracer concentration normalized by *C*_*surf*_, relative to *V*_*isopy*_ and at time *t*_2_. The dashed contour in subplot (**b**) identify the reference isopycnal surface at the initial time *t*_1_.

In Fig. 5 the effect of the secondary flow on deep ventilation is investigated by means of the results of the tracers experiments, for the (actual) rotating case. Results are presented in terms of along-lake distribution of normalized ventilation volume *η*(*i*, *t*) (defined as in (2), Fig. 5a), and maps of mean tracer concentration normalized by *C*_*surf*_ relative to *V*_*isopy*_ and at time *t*_2_ (Fig. 5b). The ventilation of the water volume below the reference isopycnal surface affects the whole northern trunk of the lake. This phenomenon is the result of lateral flows orthogonal to the wind direction, which are well distributed along the entire region (see Fig. 4c). Specifically, significant ventilation of *V*_*isopy*_ occurs between 20 km from the southernmost shore and the northern end, corresponding to about the 60% of the lake’s length. The process shows a progressive intensification in time, according to the initiation and evolution of the wind-driven secondary flows discussed above. In addition, such intensification gradually propagates from the south towards the north end of the lake, consistently with an expected along-lake circulation aligned with the wind forcing, which causes a progressive tilting of the isopycnals, with upwelling at the upwind end of the lake (north) and downwelling at the other end (south). The combined transverse (due to lateral flows, see Fig. 4a,c) and longitudinal (due to along-lake circulation) tilting of the isopycnals produces enhanced shear in the thermocline region due to vertical and lateral density gradients, which increases after wind relaxes as water masses return to new equilibrium conditions, and causes large-scale convective mixing among adjacent water masses. In the southern and wider part of the lake, no deep ventilation is observed, the bathymetry being shallower than the reference isopycnal surface (Fig. 5b).

Based on these results, we estimate that after two and four days (i.e., at *t*_2_) of persistent northerly wind with an average speed of ~6.5 m/s, about the 2% and 4% of *V*_*isopy*_ are ventilated by wind-driven mixing. Similar results are found for the March simulation (shown in the Supplementary Information), although due to the shorter and weaker wind forcing (see Fig. 2) the overall ventilation efficiency *η* after two and four days was about 1.1% and 1.3%. While the above quantification of deep ventilation efficiency depends on several factors, among which the set-up of the tracer exercise (e.g., duration, initial tracer distribution) certainly plays a role, it provides evidence of its existence and a rough estimate of the order of magnitude.

## Discussion and Conclusions

We have shown that planetary rotation is responsible for the existence of lateral temperature and chlorophyll-a gradients in the elongated and deep trunk of Lake Garda. The combined effect of rotation and closed boundaries at the shores generates a secondary circulation which leads to along-lake uniformly distributed up- and downwelling causing the observed lateral gradients. Such secondary flows were theoretically analyzed for homogeneous density conditions in elongated rotating basins^39–41^ and were hypothesized as possible deep ventilation mechanism in large lakes such as Lake Baikal^1,65–67^ where the effect of Earth’s rotation is undoubtedly present. However, their effects had so far never been explicitly observed in relatively small narrow and deep lakes, where their presence was often considered unlikely (e.g.^68^).

The occurrence of these secondary circulations has several implications for the hydrodynamics and ecosystem of lakes. In fact, they are associated with lateral but also deep vertical transport of energy and mass along the entire elongated basin, causing basin-wide DMEs that contribute to the exchange of oxygen and nutrients (but also contaminants) between surface and deep water layers. Importantly, secondary flows were observed also in stratified conditions in summer (albeit with a smaller vertical extent), indicating their year-round importance for transport processes in the lake.

While it is clear that an elongated shape favoring the development of persistent one-directional winds is a key condition for the existence of such secondary flows, more work is needed to disclose how relevant these results are for other lakes. For example, by analyzing some deep Norwegian thermobarically stratified fjord lakes^69^, found a close relationship between ventilation due to deep water formation and lake length. While wind-driven along-lake temperature gradients have been hypothesized as the possible control of deep water formation based on the relatively limited available data, we suggest that lateral temperature patterns due to the effect of planetary rotation should be considered carefully when planning future monitoring activities in those or similar narrow lakes.

The magnitude of such secondary flows and associated DMEs versus relevant parameters of a lake such as geometry, bathymetry, thermal stratification (e.g., thermobaric effects), latitude, and wind-stress forcing remains to be quantified. In this last respect, however, classical analytical solutions for finite or infinite domains exist and suggest that wind-driven lateral transport due to the Coriolis force depends on the square of the wind speed^70^, a relationship that was confirmed also in the preliminary numerical study of the transport processes in Lake Garda^54^. Improving our understanding of these processes is required to assess to what extent wind-driven ventilation and the more ordinary buoyancy-driven convective mixing combine to prevent deoxygenation and eutrophication. In this regard, it should be noted that the ventilation efficiency of buoyancy-driven DMEs is certainly larger than that resulting from wind-driven mixing, which has been estimated here. However, the observed climate-induced decrease in buoyancy-driven DMEs due to global warming^57^ suggests that the relative importance of these two processes causing DME may change in the future. In fact, despite the relatively small ventilation efficiency of an isolate wind-driven DME, intense and long-lasting wind events are abundant in the winter season in the Alpine region^52^.

In order to have a rough estimate of the potential contribution of the wind-driven DMEs, we refer to the statistics of the three events investigated here, and define as a significant event the persistence of a wind speed larger than 5 m s^−1^ (considering the 1-day moving average of the along-lake component of wind speed) for at least one day. The analysis of the historical records for the M1 meteorological station (seven years, from 2012 to 2018) evidenced that the winter period (i.e., February-April) is characterized by the occurrence of 5 significant wind events per year (median value; 25^th^–75^th^ percentiles: 4–8 events), with a duration of 34 h (median value; 25^th^–75^th^ percentiles: 29–40 h) and intensity of 5.8 m s^−1^ (median value; 25^th^–75^th^ percentiles: 5.4–6.6 m s^−1^). Thus, considering the estimates of *η* for the single March and April events, and despite the inherent uncertainty associated to them, the exchanges between surface and deep waters may be larger than expected, and in any case should be compared to absence of buoyancy-driven DMEs since 2006.

This analysis suggests that identifying all the drivers of DMEs is a necessary step towards determining the impact of climate change on the ecosystem functioning of Lake Garda, an issue that should be high on the agenda of regional policy makers, considering the environmental and economic (for tourism in particular) importance of this water resource.

## Supplementary information

Supplementary Information

## Data Availability

Water quality profiles where provided by ARPAV and are available upon request (orac@arpa.veneto.it). Wind data were provided by ARPA-Lombardia and can be downloaded from www.arpalombardia.it. Measurements and Delft3D model results and configuration files are available at https://surfdrive.surf.nl/files/index.php/s/okgUotmqxPPf8v8.
