# Supplementary material for: Importance of planetary rotation for ventilation processes in deep elongated lakes: Evidence from Lake Garda (Italy)
Source: Sci Rep. 2019 Jun 5;9:8290. doi: 10.1038/s41598-019-44730-1 (PMC6549250; doi:10.1038/s41598-019-44730-1)
Supplement: Supplementary file 1 — Supplementary Information [file 41598_2019_44730_MOESM1_ESM.pdf]

Supplementary Information for Importance of planetary rotation for ventilation processes in deep elongated lakes: Evidence from Lake Garda (Italy)

Sebastiano Piccolroaz[1,*], Marina Amadori[2], Marco Toffolon[2], and Henk A. Dijkstra[1]

[1]Institute for Marine and Atmospheric research Utrecht, Department of Physics, Utrecht University, Utrecht, 3584 CC, The Netherlands.
[2]Department of Civil, Environmental, and Mechanical Engineering, University of Trento, Trento, I-38123, Italy.
[*]sebastiano.piccolroaz@gmail.com

Introduction

This Supplementary Information provides additional details on:

- heat exchanges between lake and atmosphere during the analyzed period (Fig. S1);
- the temperature and chlorophyll-a profiles measured in different periods of the year (Fig. S2);
- validation and additional results of the numerical models (Text S1, Figs. S3- S9);
- statistics of the wind events under investigation (Fig. S10).

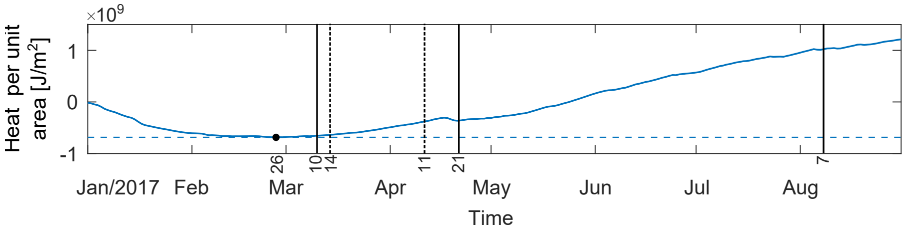

Figure S1. Time series of the cumulative heat per unit area exchanged between the lake and the atmosphere after 1 January 2017: $E(t) = \int \phi dt$, where ϕ is the net heat flux at the lake-atmosphere interface and t is time. Decreasing trends in E mean negative net heat flux to the lake (cooling), while increasing trends mean positive heat flux to the lake (warming). Winter cooling occurred until end of February (black dot), thereafter the lake started to warm up. The net heat flux ϕ was calculated using the meteorological variables from ground stations and by means of the Lake Heat Flux Analyzer code[1].

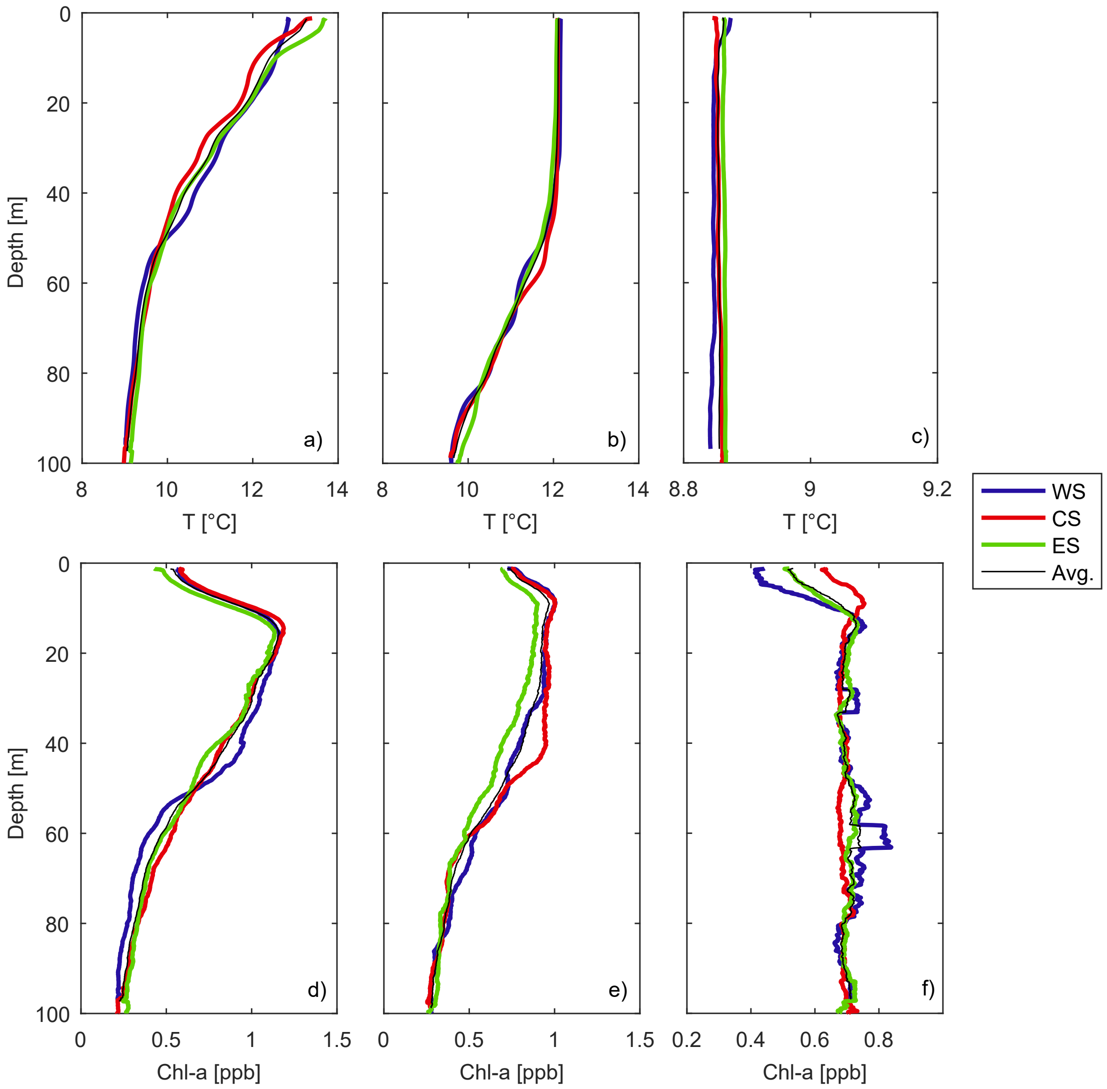

Figure S2. (a-c) Vertical profiles of temperature measured at the three sampling stations (ES, CS, and WS), on 22 May 2017, 21 November 2017, and 28 February 2018, respectively. (d-f) As in panels (a-c) but for chlorophyll-a. The profiles, measured in absence of persistent wind events, do not show any systematic lateral variability in temperature or chlorophyll-a.

Text S1: Numerical models

According to the field campaign dates (10 March, 21 April, and 7 August 2017), numerical simulations of atmospheric and lake conditions were performed for the periods 3-11 March, 18-21 April, 1-15 August 2017. The value of the main calibration parameters of the model are summarized in Table 1 in the main text. In the main text we present and discuss the results from the April event. Results for the events on March and August 2017 are shown in Figs. S3 and S4 in terms of maps of surface water temperature. Results of the tracer experiment for the event on March 2017 are shown in Fig. S5.

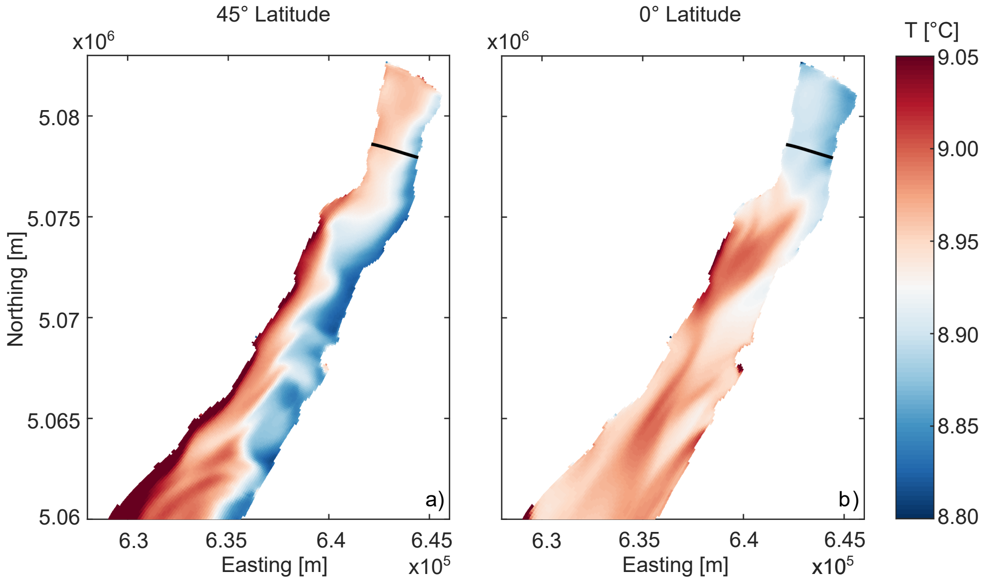

Figure S3. Results of thermo-hydrodynamic numerical simulations for the wind event on March 2017: map of surface water temperature accounting for (a) and excluding (b) the Coriolis force. Numerical results are averaged over a 6-hour period between 06:00 and 12:00 on 10 March 2017.

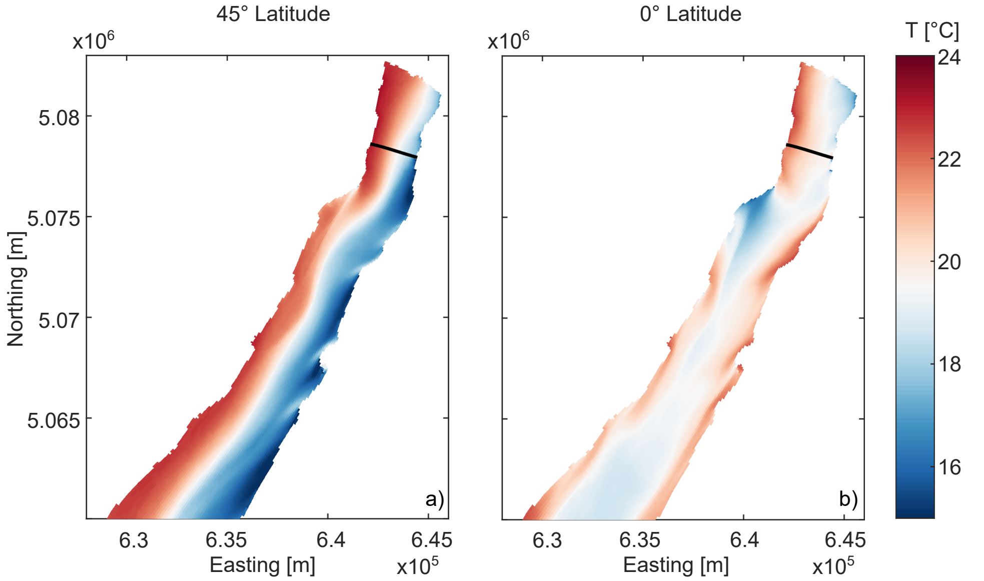

Figure S4. Results of thermo-hydrodynamic numerical simulations for the wind event on August 2017: map of surface water temperature accounting for (a) and excluding (b) the Coriolis force. Numerical results are averaged over a 6-hour period between 00:00 and 06:00 on 7 August 2017.

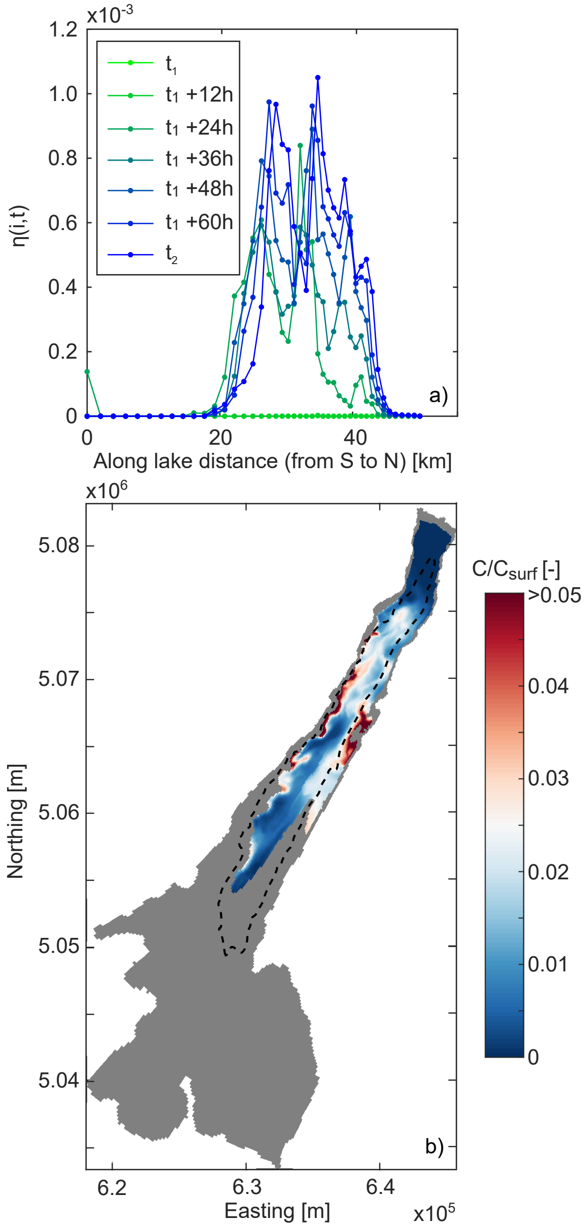

Figure S5. Results of the numerical tracer experiment for the wind event on March 2017. (a) Along-lake distribution of the normalized ventilation volume η from the release of the tracer (t_1) to the end of the experiment (t_2). (b) map of tracer concentration normalized by C_{surf}, relative to V_{iso} and at time t_2. The dashed contour in subplot (b) identify the reference isopycnal surface at initial time t_1.

Equilibrium simulations

In addition to the event-based simulations, we verified the development of secondary circulations also by performing long-term simulations. Long-term hydrodynamic simulations were performed using available meteorological fields covering a 10-year period (2003-2014), and used to generate a wind atlas for the Trentino region[2]. In this case, the final horizontal resolution of the model was 2 km and variables were saved at a temporal resolution of 1 hour.

For the hydrodynamic simulations, a coarser grid was used, with 64×224 cells of horizontal resolution varying from ∼100 m to ∼400 m. The vertical discretization and turbulence settings were the same as in the event-based simulations case. The computational time step was fixed to 20 seconds. The simulations were performed replicating the 2004 forcing (available from[2]) for six consecutive years, in order to remove the influence of initial conditions from the hydrodynamic simulations. Results in terms of maps of surface water temperature (Fig. S6) are fully consistent with the event-based simulations, indicating that the effect of Earth's rotation on the secondary circulation holds also in equilibrium lake flows.

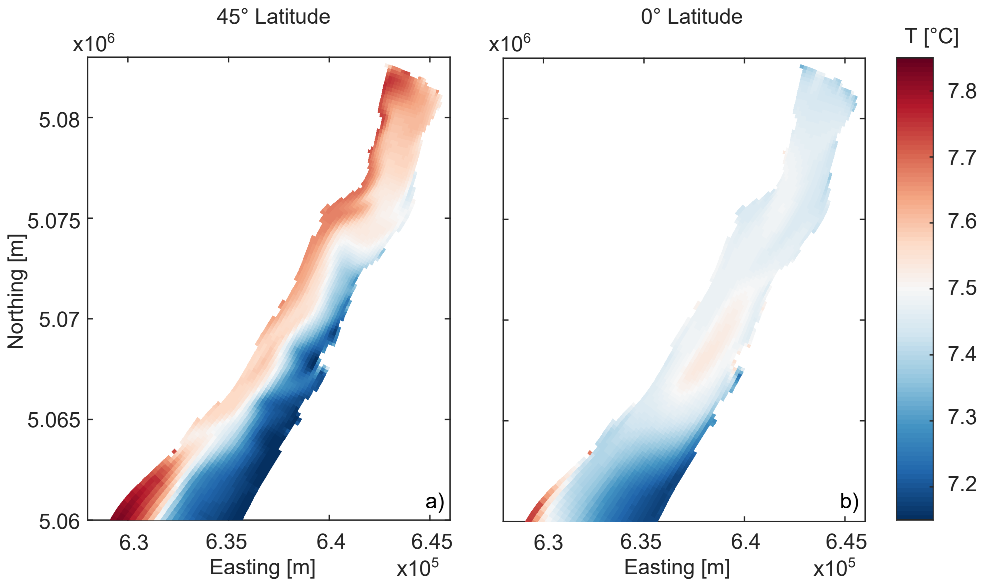

Figure S6. Results of thermo-hydrodynamic numerical simulations for the long-term simulation on March 2004 (meteorological forcing provided by the wind atlas for the Trentino region,[2]), after a persistent northerly wind event: maps of surface water temperature accounting for (a) and excluding (d) the Coriolis force. Numerical results are averaged over a 6-hour period between 12:00 and 18:00 on 23 March 2004. Coherent with the results obtained running event-based simulations, a clear accumulation of warm surface water is observable along the west shore (to the right of the wind), while cold upwelling water is visible at the east coast.

Validation of the models

In this section, we present the validation of the atmospheric and hydrodynamic models by comparing observed and simulated wind forcing and lake thermal profiles for the event-based simulations.

The validation of the atmospheric model was performed comparing observed wind roses at the Limone del Garda-M1 meteorological station (Fig. S7a, b, and c) with simulated wind roses extracted from the WRF lake cell closest to that station (Fig. S7g, h, and i). The comparison shows a very good match between simulated and observed wind direction, confirming that the model well captures the predominant winds during the three analyzed events. Concerning wind intensity, simulated values (Fig. S7g, h, and i) are generally higher than observed (Fig. S7a, b, and c). The main reasons for this difference are due to the different height at which the wind is referenced and to the small-scale factors affecting ground observations (e.g., trees, buildings, local morphology) that are partially filtered out by the 1 km spatial resolution of the WRF model. Moreover, wind speed measurements collected at ground stations do not necessarily represent over lake conditions. By comparing the wind speed data measured at the Limone meteorological station and by a portable meteorological station installed on the boat during fieldwork, we found that the wind speed above the lake was on average 1.6 times the one measured by the ground station ($WS_{lake} = 1.6WS_{land}$). This proportionality was used to rescale land-based measurements In all cases, wind speed values were converted to the standard height of 10 m for fair comparison (the same height above ground of WRF results). This is just a rescaling of wind intensity and hence cannot obviate the inherent differences between over lake and over ground wind patterns that certainly exist. However, the improved comparison between rescaled observations (Fig. S7d, e, and f) and WRF results

provides evidence that WRF results are representative of the lake forcing conditions. Additional and systematic validation of the WRF model is provided in[2] and[3], which used the same setup of the WRF model to simulate thermally-driven circulations in the lake area and surrounding territory.

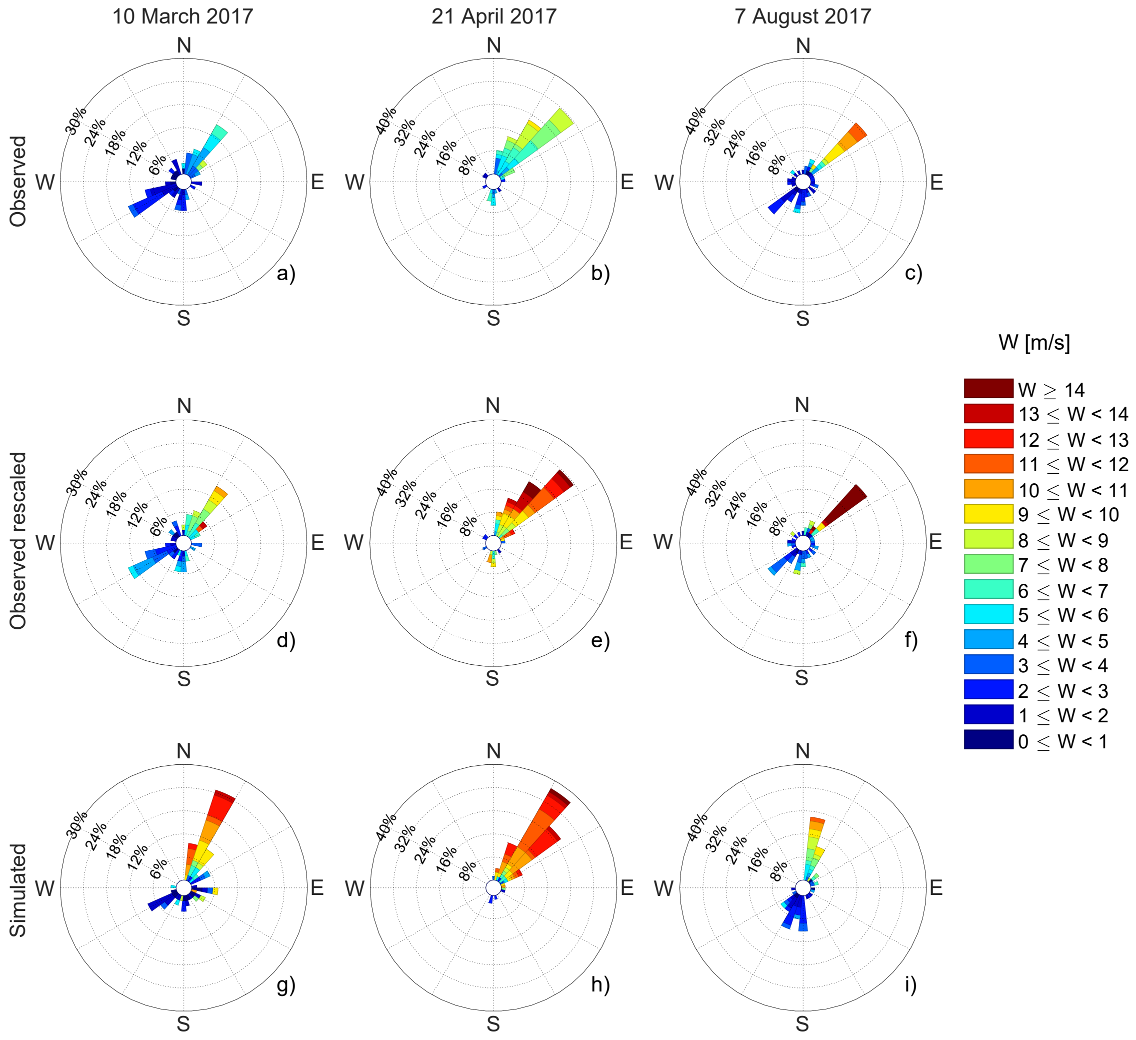

Figure S7. Wind roses for the three analyzed periods based on measurements collected at 16 m above ground at station M1 (see Fig. 1 a) (a-c), measurements collected at station M1 referred to 10 m above ground and rescaled according to $WS_{lake} = 1.6 WS_{land}$ (d-f), and WRF simulations at 10 m above ground (g-i). Wind roses refer to a three-day period, including the fieldwork day and the two preceding days.

The hydrodynamic model was validated by comparing simulated and observed vertical temperature profiles along the reference transect. A one-to-one comparison for the three analyzed periods is provided in Fig. S8. Considering the uncertainties in the meteorological forcing and given the limited data for model calibration, the results show a high degree of coherence with observations in terms of stratification and lateral variability. In general, the RMSE between simulations and measurements in the upper 100 m is small and on the order of 0.01 °C in March, on the order of 0.1 °C in April, and less than 1 °C in August. These values of RMSE are fully compatible with model performances in other studies conducted with the use of Delft3D (e.g.[4,5]). The positioning of the deepest thermocline is also well simulated at the western station (WS, 60-70 m in March, 40-50 m in April, 10 m in August), and the more homogeneous conditions at the eastern station (ES) are correctly reproduced. While we acknowledge that the model slightly underestimates temperature and stratification in April, we notice that the lake was very weakly stratified in March and April, which certainly hampered the simulation of the temperature profiles. Observed vertical temperature and density gradients were about 0.01 $°C m^{-1}$ and 5×10^{-3} kg m^{-3} m^{-1}, substantially lower - one to two order of magnitude - than the typical thresholds used to identify the well mixed layer (e.g.[6]).

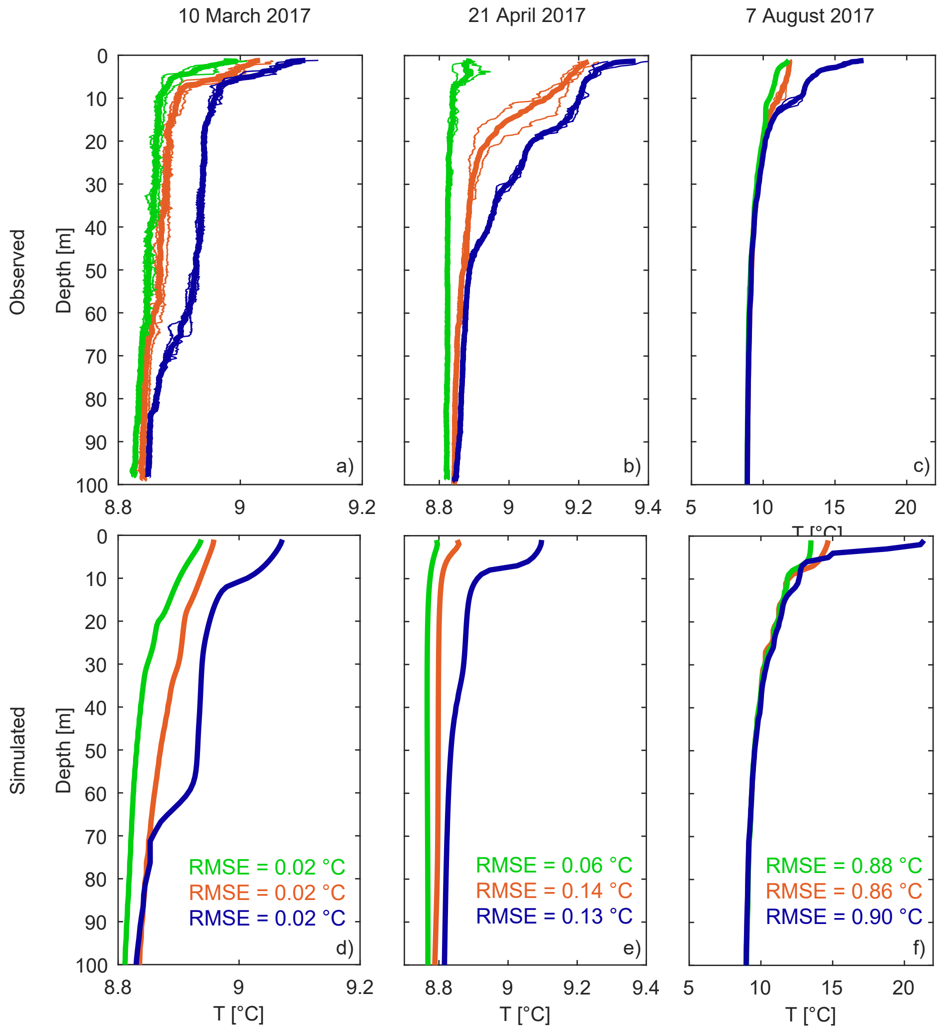

Figure S8. Vertical profiles of temperature measured at the three sampling stations (ES, CS, and WS) in the three analyzed periods (a-c) and simulated by the Delft3D model (d-f). The Root Mean Square Error (RMSE) between simulated and observed profiles is also shown.

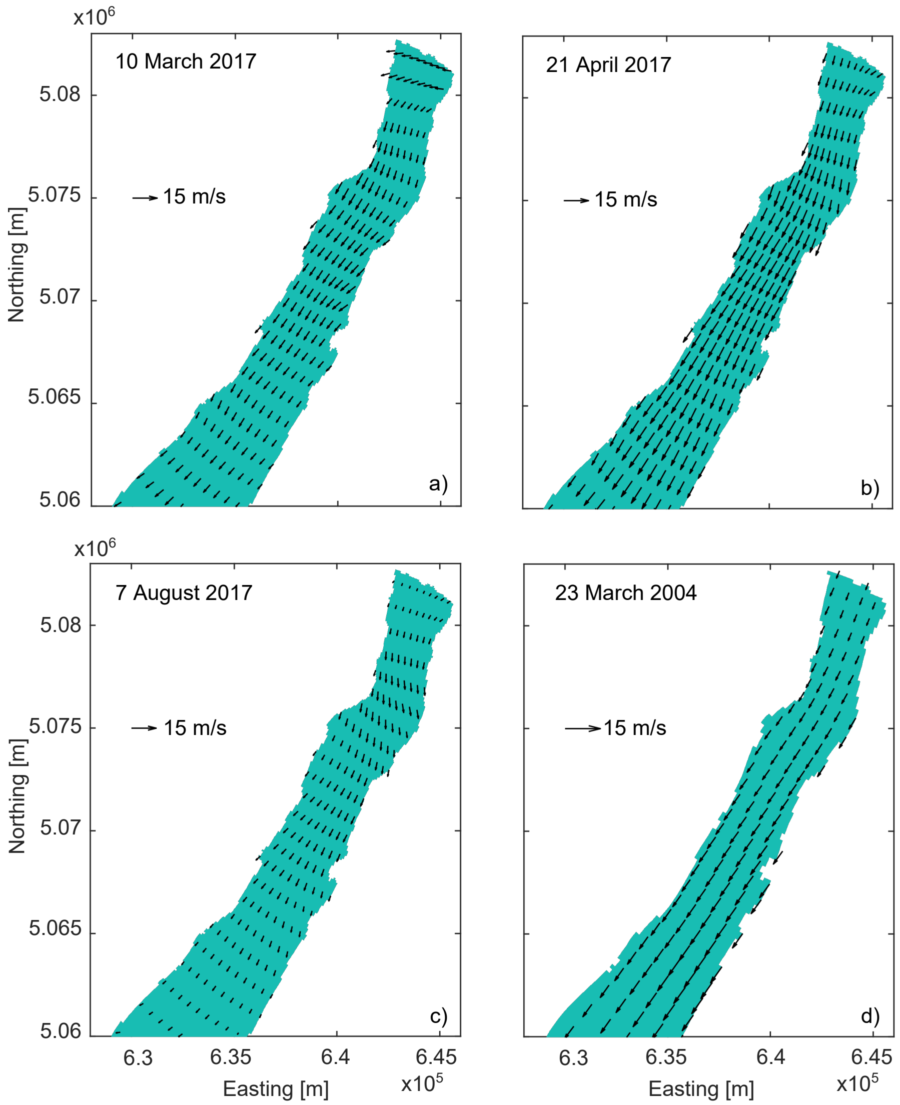

Figure S9. Wind fields simulated by the WRF model on 10 March 2017 (a), 21 April 2017 (b), 7 August 2017 (c), and 23 March 2004 (d). Wind fields have been averaged over a 6-hour period coherently with the hydrodynamic results shown in the corresponding Figs. 4 (in the main text), S3, S4, and S6.

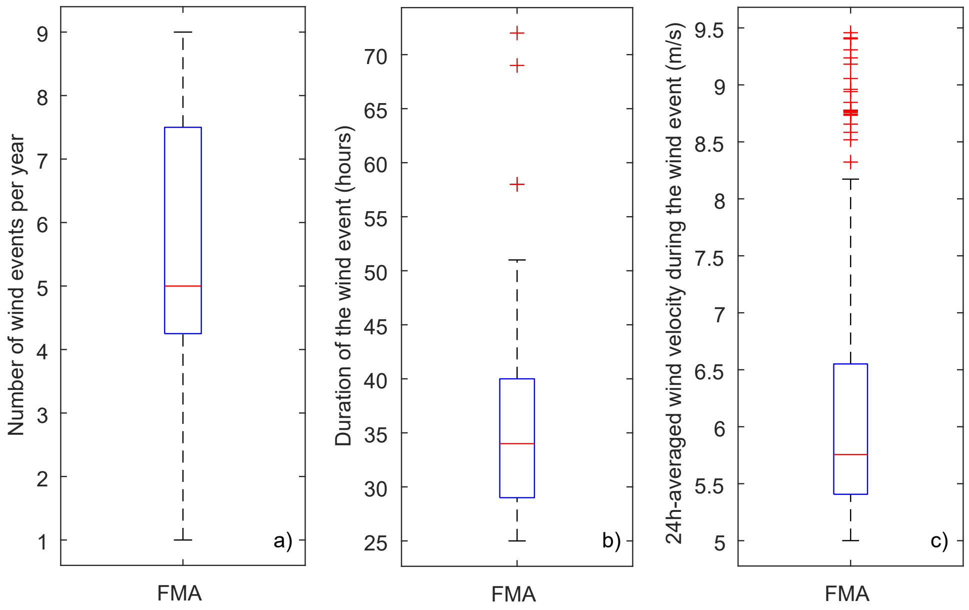

Figure S10. Statistics of significant wind events, defined as persistent events with the along-lake component of wind speed larger than 5 m s^{-1} (considering the 1-day moving average) for at least one day: a) number of events occurring per year; b) duration of the wind events; c) wind velocity along the longitudinal axis of the lake. Statistics refer to the meteorological stations in Limone del Garda-M1 during the weak stratification period (February to April).

References

1. Woolway, R. I. *et al.* Automated calculation of surface energy fluxes with high-frequency lake buoy data. *Environ. Model. & Softw.* **70**, 191 – 198, DOI: https://doi.org/10.1016/j.envsoft.2015.04.013 (2015).
2. Giovannini, L., Antonacci, G., Zardi, D., Laiti, L. & Panziera, L. Sensitivity of simulated wind speed to spatial resolution over complex terrain. *Energy Procedia* **59**, 323–329, DOI: 10.1016/j.egypro.2014.10.384 (2014).
3. Giovannini, L., Zardi, D., de Franceschi, M. & Chen, F. Numerical simulations of boundary-layer processes and urban-induced alterations in an Alpine valley. *Int.l J. Clim.* **34**, 1111–1131, DOI: 10.1002/joc.3750 (2014).
4. Razmi, A. M., Barry, D. A., Bakhtyar, R. & Wüest, A. Current variability in a wide and open lacustrine embayment in Lake Geneva (Switzerland). *J. Gt. Lakes Res.* **39**, 455–465, DOI: 10.1016/j.jglr.2013.06.011 (2013).
5. Wahl, B. & Peeters, F. Effect of climatic changes on stratification and deep-water renewal in Lake Constance assessed by sensitivity studies with a 3D hydrodynamic model. *Limnol. Oceanogr.* **59**, 1035–1052, DOI: 10.4319/lo.2014.59.3.1035 (2014).
6. Read, J. *et al.* Derivation of lake mixing and stratification indices from high-resolution lake buoy data. *Environ. Model. Softw.* **26**, 1325 – 1336, DOI: 10.1016/j.envsoft.2011.05.006 (2011).
